# 

**Published:** 1891-12

**Authors:** 


### HALL'S JOURNAL OF HEALTH

TRUTH DEMANDS NO SACRIFICE; ERROR CAN MAKE NONE.

Vol. 38. DECEMBER, 1891. No. 12.

### SPECIAL.

The demand for back volumes of our JOURNAL has been so great that we very much require a few complete sets of the year 1888.

If any of our subscribers are able to supply us with them, we should esteem it a great favor if they would signify the fact by a postal card, stating price.

### THE NEXT VOLUME.

The 39th volume of our JOURNAL will begin with the new year. Those who have paid their dollar for it in the past, will not be likely to give it up now, for truth to say, the dollar has been saved over and over again by following some course of preventive or cure recommended in its columns. But credit is seldom awarded to the publication whose forewarnings operate as a defense against the inroads of disease, there are so many ways of shifting and avoiding such a confession. It is nevertheless true that very many diseases that "flesh is heir to" may be avoided by a reasonably correct system of living. This includes pure air, wholesome food, open air exercise and temperance in all things. Very many who condemn in unmeasured terms the habitual wine bibber, are slowly undermining their health by over eating. Indeed, it is a grave question which of the two evils is responsible for the greater number of premature deaths.

It is only necessary to review the pages of HALL'S JOURNAL OF HEALTH to learn with what persistency it has enforced upon its readers the absolute necessity of avoiding either extreme. But in spite of every precaution disease will make frequent inroads upon the best appointed homes, owing in great measure to the prevalence of various corrupting influences which pervade the atmosphere, against which it is ordinarily impossible to guard. In such instances the assistance of a skillful physician becomes clearly necessary, and this, too, in the very first stages of the malady. Although this JOURNAL does not advocate the free use of drugs, especially such as are extracted of mineral substances, there may be occasions when their employment amounts to a saving necessity. Of such, we do not assume to judge. Our aim has been to impart such information as may be specially valuable to the household in its several departments, and to keep its advertising columns not only clean of objectionable matters, but so served as to furnish something of a guarantee of the value of the articles to which attention is thus directed. If others of a questionable nature, by any trick or artifice, have been admitted, on being discovered they have been summarily ejected, with an invitation not to call again.

In a word, we mean to deal justly and uprightly with our patrons of whatever class or degree.

To such of our subscribers, old and new, as remit their one dollar subscription directly to this office, we offer a selection of premiums which ought to induce a liberal patronage, many of them being equal in value to the whole remittance. Our limited space will not admit of publishing a complete list of works available to subscribers for this purpose, but one can scarcely go amiss in ordering as a premium almost any miscellaneous work of well known authors contained in a single volume.

### LITERARY.

History of Circumcision, from the Earliest Times to the Present, etc. By P. C. Remondino, M.D. F. A. Davis, publisher, Philadelphia. 346 pp. Price, cloth, $1.25 ; paper, 50c. net.

The scope of this work embraces a much wider range than its title would indicate. It deals not only with circumcision, as practiced by the Egyptians in pre-historic times, but follows the custom as incorporated into the rites and practices of other Eastern peoples, first as a sanitary, and finally, a religious measure, but gives, with historical data and detail, the introduction among various nations and tribes for various causes, prudential and penal, of the complete emasculation of the procreative property in males, going learnedly and with great thoroughness into the subject, from remote periods to the present day.

In its special department the work has great value, and is equally adapted to the medical profession and the general scholar.

Effects of Massage ; compiled by Leroy Henry, Practical Masseur, St. Louis, Mo. Price 10 cents.

Conveys much useful information upon a system of treating invalids which has obtained great popularity within the past few years.

Trifet's Monthly Galaxy of Music, for November. Send one dime for it, 408 Washington street, Boston.

As full of good things as the enterprising publisher after his Thanksgiving dinner.

Experiments with Sewage and Waste-pipe Traps, with a View to the Efficiency of Various Appliances, with Tabulated Results and Illustrations. By James M. Denton, M. E. 56 pp.

Reprinted from Transactions of American Public Health Association.

Papers in Penology.—

This is a handy little volume of some 150 pp., which treats of reformatory institutions, prisons and prison systems, and more particularly of the Elmira Reformatory of New York, which has held its place for so many years as one of the best institutions of the State. The several chapters are a compilation from different writers, able, interesting and instructive. The article upon "The Prisons of Great Britain," states that "The officers enter the service in the lowest positions," and secure promotion solely by their individual merits, without the influence of "Poitical patronage or private interest." How does that strike you, Governor ?

Ethereal Matter, Electricity and Akasa. By N. Kolkin. For sale by The J. M. Pinckney Book and Stationery Co., Sioux City, Iowa. Price 50 cents.

Akasa is here described to be either matter or a condition of matter having relation to the soul or ultimate life principle. The chapters on this subject are highly interesting, and show that

"There are more things in heaven and earth, * * *
Than are dreamt of in our philosophy."

ALL AROUND THE YEAR; LEE AND SHEPARD'S ORNAMENTAL CALENDAR FOR 1892. Lee & Shepard, publishers, Boston. Price 50 cents.

Well, there's no use trying to beat it, so it is better to give it up in the beginning than to fail in the end. In elegance of design, richness of material and artistic adornment, it takes the lead. There are fourteen beautifully illustrated cards, bound in a silver chain, embracing the 12 calendar months, in themselves a "thing of beauty;" and if not "a joy forever," it is no fault of the publishers.

Since writing the above we have made arrangements to offer this beautiful annual as a premium to the subscribers of this JOURNAL.

We are under obligations to the several authors for the following valuable treatises:

ELECTRICITY IN CARCINOMA. By Robert Newman, M. D., New York, Consulting Surgeon to Hackensack and Bayonne Hospitals, etc.

This paper maintains that *cancer* is *curable* by the electrical process, and cites instances of cure in detail.

TUMORS OF THE NASO-PHARYNX, LARYNX AND ÆSOPHAGUS. By W. Cheatham, M. D., of the University of Louisville.

THE STATISTICS AND LESSONS OF 1500 CASES OF REFRACTION. By George M. Gould, M. D., Philadelphia.

A PLEA FOR THE EXTRA-PERITONEAL TREATMENT OF STUMP IN ABDOMINAL HYSTERECTOMY FOR FIBROIDS. By S. Laptham Smith, M. D., etc., Toronto, Canada.

THE CENTRAL UNIVERSITY OF KENTUCKY (Louisville), embracing Medical and Dental Departments. Announcement of 1892.

TWENTY-SEVENTH ANNUAL REPORT OF THE SHELTERING ARMS, New York City. One of the worthiest charities of this great Metropolis, where homeless girls and boys are cared for and trained in pursuits of profitable industry.

